# Postural Balance in Relation with Vision and Physical Activity in Healthy Young Adults

**DOI:** 10.3390/ijerph19095021

**Published:** 2022-04-20

**Authors:** Roxana Ramona Onofrei, Elena Amaricai

**Affiliations:** Department of Rehabilitation, Physical Medicine and Rheumatology, Research Center for Assessment of Human Motion, Functionality and Disability, “Victor Babes” University of Medicine and Pharmacy Timisoara, 300041 Timisoara, Romania; amaricai.elena@umft.ro

**Keywords:** postural balance, young adults, physical activity, stabilometry

## Abstract

Postural balance is an essential part of a wide range of activities, from daily living tasks to sports. Regularly repeated physical and/or sport activities improve both the postural performance and the postural strategy. The aim of our study was to evaluate if the physical activity level is a factor that influences postural balance performance, including the impact of vision and gender, in healthy young adults. Postural balance was assessed in 78 subjects (38 males and 40 females, aged 20.64 ± 1.18 years) by using the PoData system, in open (EO) and closed (EC) eye conditions. Based on the physical activity level, subjects were classified in two groups—low physical activity level (*n* = 36, 46.15%) and moderate physical activity level (*n* = 42, 53.85%). A group significant difference was found only for the average centre of pressure (CoP) deviations on the latero-lateral axis (CoP_X_), with a higher lateral deviation of the CoP (toward right) in the low physical activity group (F = 4.005, *p* = 0.04). CoP path length, the 90% confidence ellipse and maximum CoP speed were significantly increased in EC conditions. A statistically significant interaction effect (vision × physical activity) was observed for the CoP path length (F = 7.9, *p* = 0.006).

## 1. Introduction

Postural balance is an essential part of a wide range of activities, from daily living tasks to sports, by maintaining the centre of gravity over the support base [1]. Maintaining an upright stance is a complex task that depends on several factors related to vestibular, somatosensory and visual systems, as well as to motor abilities [2].

Studies are mainly focused on the elderly, aiming to identify factors related to poor postural balance and to evaluate outcomes of different prevention strategies for reducing the risk of falls [3,4,5,6]. In young adults, research is generally targeted to assess the influence of various factors (e.g., anthropometric characteristics, gender, body mass index, dual-tasks or participation in diverse sports) on balance [1,7,8,9,10,11,12]. A poor postural control has been suggested to play a role in the onset of non-contact lower limb injuries [13,14]. Poor postural control performance has been associated with lateral ankle instability [15]. Previous research pointed out that postural balance in healthy individuals decreases over time; conditions of support and vision can negatively affect groups of older age [16].

The beneficial role of physical activity in reducing chronic diseases is well known, as well as the detrimental effects of low physical activity or sedentarism on the musculoskeletal system, body composition, metabolic and cardio-vascular health [17,18,19]. While there are studies analysing the impact of physical activity levels and exercise on balance in older adults [20,21,22,23,24,25,26], only a few addressed this feature in healthy young adults [27,28,29,30].

Regularly repeated physical and/or sport activities improve both the postural performance and the postural strategy [31]. Kiers et al. [32] reviewed 39 studies and demonstrated that in general, sports practitioners sway less than controls in an unperturbed stance. Zhu et al. [30] reported that static balance was significantly associated with physical activity and also with sex and visual contribution. In their study, Petroman et al. [27] found that physically active young adults had significantly better postural balance than sedentary ones, with no gender-specific differences. Vision, sport specific postures and frequency and duration proved to be important determinants of the effects sports activity has on postural stability in standing [32]. Maitre and Paillard [33] found no differences in postural balance between active and non-active participants. A possible explanation was that the bipedal stance with eyes open was a too simple postural task to highlight the differences between active and non-active participants. These things considered, the active participants could have improved their dynamic balance control rather than static balance by participating in physical activities [33].

The differences in postural balance between sexes are not consistent. Previous research showed either no differences in postural sway between sexes [34,35,36], either a lower postural stability in female [9,37] or in male young adults [29,30].

Vision plays an important role in balance control, regardless of age, as it provides information of position and direction in space, and also about the environmental constraints [38,39,40,41]. Schmidt et al. [38] found that in the eyes open condition the postural control was better than in visual deprivation conditions, such as eyes closed, blackout glasses or dark room. Thompson et al. [42] found that non-athletes had greater difficulty balancing when vision was limited. In their study, Torres et al. [29] found that vision had a greater influence on balance control among sedentary men compared to sedentary women. On the other hand, expert athletes in different sports have been reported to rely less on vision for postural balance control, dedicating their visual attention to their athletic activity [43,44].

The aim of our study was to evaluate if the physical activity level is a factor that influences postural balance performance, including the impact of vision and gender, in healthy young adults. We hypothesized that the level of physical activity would influence the postural balance, physical active subjects having a better postural balance performance.

## 2. Materials and Methods

### 2.1. Participants

Eighty-two healthy young volunteer subjects agreed to participate in the study. They were recruited among our university’s graduates, their relatives, friends or acquaintances. The inclusion criteria were: aged between 18 and 25 years; with no history of lower limb or trunk injury, surgery, musculoskeletal, neurologic or vestibular disorders that could influence posture and balance. Participation in the study was voluntary. Written informed consent was obtained from all participants. The study was carried out in accordance with the Declaration of Helsinki and was approved by Ethics Committee of “Victor Babes” University of Medicine and Pharmacy Timisoara, Romania (no 23/2019).

### 2.2. Assessments

The physical activity level was assessed using the short form of the International Physical Activity Questionnaire (IPAQ). IPAQ short form (IPAQ-SF) records the physical activity in the last 7 days, at four intensity level—vigorous intensity activities (aerobics, heavy lifting, fast bicycling for at least 10 min at a time), moderate intensity activities (bicycling at regular pace, carrying light loads, double tennis for at least 10 min at a time), walking (for at least 10 min at a time) and sitting (on a weekday). The total score of IPAQ-SF represents the summation of the duration in minutes and frequency in days of walking, moderate intensity and vigorous intensity activities [45]. Based on the responses, the physical activity was ranked as low if no activity or some activity, but not enough to meet the other categories, was reported. The physical activity was ranked as moderate if 3 or more days of vigorous intensity activity of at least 20 min/day or 5 or more days of moderate intensity activity and/or walking of at least 30 min/day or 5 or more days of any combination of walking, moderate intensity or vigorous intensity activities achieving a minimum of at least 600 MET-min/week was reported. A high level of physical activity was considered if vigorous activity was performed on at least 3 days and accumulating at least 1500 MET-min/week or 7 or more days of any combination of walking, moderate intensity or vigorous intensity activities accumulating at least 3000 MET-min/week [45]. Previous studies reported that IPAQ-SF is a suitable physical activity measurement tool for young adults, with higher correlations and levels of agreement with accelerometry [46,47].

Postural balance was assessed with the PoData system (Chinesport, Udine, Italy) through stabilometric analysis. The PoData device is a capacitive pressure distribution system with an integrated podoscope and six load cells. The sampling frequency for data recording is 100 Hz [48]. Data were analysed with the GPS5 software. The recorded stabilometric data were related to the body centre of pressure (CoP). The average CoP deviation from the theoretical reference on latero-lateral (CoP_X_) and antero-posterior (CoP_Y_) axes were provided by the software [48,49,50]. A positive value represents a right deviation on the latero-lateral axis, and an anterior deviation on the antero-posterior axis [51]. The absolute mean CoP displacement from the ideal position was calculated, based on the deviations on antero-posterior and latero-lateral deviations [51,52]. The centre of gravity shift during the test was measured and reported as the CoP path length. The confidence ellipse area was measured in mm^2^, as the area of the ellipse that includes all the centre of gravity points measured and transferred on a Cartesian system of axes with 90% level of confidence [51]. The average centre of gravity shifting maximum speed was measured and reported as maximum CoP speed. For the postural assessment, the subjects had to stand on the PoData platform in an upright posture, with lower limbs extended and arms positioned naturally along sides, barefoot, with the feet positioned at an angle of 30° to each other and 5 cm between heels [51,53]. The postural assessments were performed in two conditions—with eyes opened and eyes closed. For each condition, the assessment had a duration of 20 s, one trial per condition being performed.

### 2.3. Statistical Analysis

Statistical analysis was performed using SPSS version 28.0.1.1 (IBM Corporation, Armonk, NY, USA). Data were tested for normality using Shapiro-Wilk test and descriptive statistics was performed (mean and standard deviation for normal distributed data and median and interquartile range [IQR] for non-normal distributed data). Data were analysed using a repeated measures general linear model, with vision (eyes open, eyes closed) as the within-subjects factor and physical activity level (low physical activity, moderate physical activity) and gender (female, male) as between-subjects factors. Bonferroni corrections was applied for multiple comparisons. Statistical significance was set *p* < 0.05 for all tests.

## 3. Results

Seventy-eight subjects (38 males and 40 females, aged 20.64 ± 1.18 years) met the inclusion criteria and agreed to participate in the study. Four participants did not meet the inclusion criteria (two participants reported an ankle sprain in the last month, one participant had a partial anterior cruciate ligament and one participant could not attend the assessment session). Based on the physical activity level, subjects were classified in two groups—low physical activity level (*n* = 36, 46.15%) and moderate physical activity level (*n* = 42, 53.85%). The demographic characteristics are presented in Table 1.

The stabilometric data are presented in Table 2.

The within-subjects factor (vision: eyes open/eyes closed) and between-subjects factors (physical activity level: low/moderate physical activity; gender: male/female) and the interactions of these factors are presented in Table 3 for all stabilometric data.

A group significant difference was found only for the average CoP deviations on latero-lateral axis (CoP_X_), with a higher lateral deviation of the CoP (toward right) in the low physical activity group (F = 4.005, *p* = 0.04).

**Figure 1:**
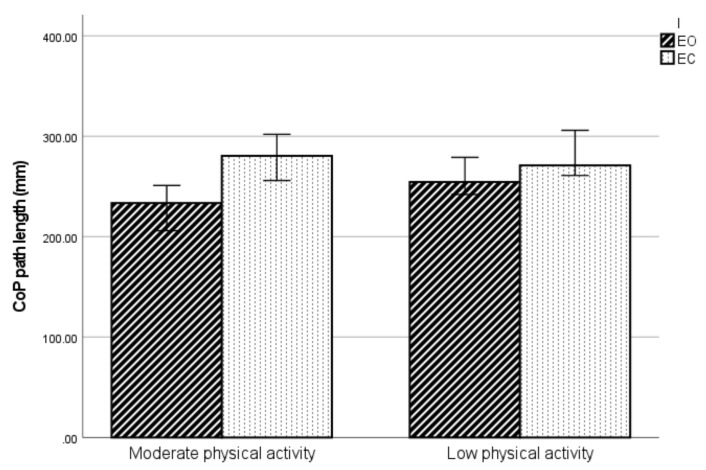
Interaction effect (vision × physical activity) on CoP path length.

CoP path length, the 90% confidence ellipse and maximum CoP speed were significantly increased in EC conditions (vision effect—CoP path length: F = 49.3, *p* < 0.001; 90% confidence ellipse: F = 5.94, *p* = 0.01; maximum CoP speed: F = 19.78, *p* < 0.001). A statistically significant interaction effect (vision × physical activity) was observed for the CoP path length (F = 7.9, *p* = 0.006) (Figure 1).

## 4. Discussion

The current study aimed to investigate the relationship between physical activity level, vision and gender and postural balance in healthy young adults. We compared CoP parameters in healthy young adults with different physical activity levels, both in eyes open and closed conditions. Our findings showed that postural balance was influenced by the interaction of physical activity level and vision in healthy young adults. Moreover, the postural balance was influenced by the visual contribution, with no relation with the physical activity level of healthy young adults. No gender differences were found in postural balance performance in healthy young adults.

CoP path length quantifies the magnitude of the two-dimensional displacement based on the total distance travelled; a smaller CoP path length value implies a better postural stability [54]. Our results showed that low physical activity levels in the absence of visual information determined a significant increase in CoP path length. When compared to subjects with moderate physical activity, healthy young adults with low physical activity had a poor postural balance (expressed as the latero-lateral deviation of the body centre of pressure).

In their study, Zhu et al. [30] found that increased moderate-to-vigorous physical activity or less sedentary time was associated with a better performance in static balance in young adults aged 18–26 years. They have objectively assessed physical activity using accelerometers and found that only moderate-to-vigorous physical activity level and sedentary time were significantly associated with sway area.

Orofino et al. [2] investigated the relation between postural stability and various types of physical activities training among university students. They found that students who played balance-based exergames almost two times per week had better postural stability performance than those participating in different sports (Sports) or not participating in any type of physical activities (Control). The research showed significant differences for any posturographic parameters between the Sport and Control group, in either eyes opened or closed condition [2]. A possible explanation was that the sports played by the subjects involved in the study were not oriented to balance abilities. A similar conclusion was reported by Jakobsen et al. [11], who found superior changes in postural stability after 12 weeks of soccer training compared with high-intensity running or continuous running training in untrained men, aged 21–45 years.

In their study, Andreeva et al. [55] showed that practicing any kind of sport was associated with increased postural stability in normal bipedal stance. The authors stated that practicing any sports could have a favourable nonspecific effect on posture stability through the improvement of sensory, muscular and integrative central nervous components of the posture regulation system [55]. Zhu et al. [30] also suggested that physical activity contributes to balance gains by stimulating muscular strength and endurance of the lower and upper limbs, and also by promoting neurophysiologic adaptations that increase reflex responses and proprioception.

In the EC conditions, a significant increase in other COP parameters (CoP path length, maximum CoP speed and 90% confidence ellipse) were reported, highlighting the importance of vision on postural balance in healthy young adults. Our results are in accordance with those reported by Grace Gaerlan et al. [39], who stated that the visual system was the predominant sensory system used by young adults to maintain optimal postural balance. Orofino et al. also found better scores for all posturographic parameters in the open eye test than in the closed eyes test [2]. Similar to our results, Zhu et al. [30] found that participants in their study maintained a more stable position with the eyes opened, having significant lower values for sway path length, sway area and sway velocity.

A practical application that results from the findings of our study is that the increase in physical activity level (even though not reaching a high activity level) will lead to a better static postural balance. Thus, those subjects usually involved in different sport or recreational activities will have a lower risk of musculoskeletal lower limb injuries. Maintaining an adequate postural balance in subjects throughout the life course is a part of the prevention strategy for healthy ageing and well-being.

Some limitations of this study have to be considered in interpreting the results. We only assessed young adults, aged 18–25 years. The lack of a high physical activity level group is another limitation. This study focused on the assessment of static postural balance; the dynamic balance was not tested due to a lack of the necessary equipment. Further studies are needed to evaluate the impact of all levels of physical activity in different age groups, as well as the assessment of both static and dynamic postural balance.

## 5. Conclusions

Our study assessing healthy young adults points out that the level of physical activity influences postural balance. Postural balance was influenced by the interaction of physical activity level and vision in healthy young adults. Moreover, the postural balance was influenced by the visual contribution, with no relation with the physical activity level of healthy young adults. No gender differences were found in postural balance performance in healthy young adults. Increase in physical activity level in healthy subjects should be targeted as this category of the population is frequently involved in domestic and professional tasks, as well as in leisure sports activities that need a good postural balance.

## Figures and Tables

**Table 1:** Subjects’characteristics.

	Low Physical Activity Group (*n* = 36)	Moderate Physical Activity Group (*n* = 42)	All (*n* = 78)	*p* *
Age, years (mean ± SD)	20.56 ± 1.18	20.71 ± 1.19	20.64 ± 1.18	NS
Gender				
Female, *n* (%)	19 (52.78)	21 (50)	40 (51.28)	NS
Male, *n* (%)	17 (47.22)	21 (50)	38 (48.72)	
Weight, kg (mean ± SD)	73.55 ± 12.93	62.56 ± 9.44	67.63 ± 12.41	<0.0001
Height, cm (mean ± SD)	172.6 ± 8.32	172.5 ± 7.95	172.5 ± 8.07	NS
BMI, kg/m^2^ (mean ± SD)	24.61 ± 3.62	20.95 ± 2.34	22.64 ± 3.5	<0.0001

* *p* relates to between groups differences; NS – non significant.

**Table 2:** Stabilometric data.

	Low Physical Activity Group (*n* = 36)		Moderate Physical Activity Group (*n* = 42)		All (*n* = 78)	
	Eyes Open	Eyes Closed	Eyes Open	Eyes Closed	Eyes Open	Eyes Closed
CoP_X_, mm (median [IQR])	4.5 [−3–8]	3 [−3.5–9]	0 [−6–5]	−0.5 [−7–4]	2 [−5–7]	1 [−6–6]
CoP_Y_, mm (median [IQR])	4.5 [−2–16.5]	10 [−1.5–17]	4.5 [−4–16]	3 [−3–17]	4.5 [−3–16]	6 [−2–17]
COP displacement, mm (median [IQR])	13.43 [8.83–21.32]	14.4 [10.99–19.98]	13.91 [8.06–20.59]	13.19 [7.61–19.64]	13.67 [13.67–21.09]	13.72 [10.05–19.64]
CoP path length, mm (median [IQR])	254 [228–298]	271 [247–322]	233.5 [200.5–259.5]	280.5 [249–308]	245 [211–278]	279 [248–320]
90% Confidence ellipse, mm^2^ (median [IQR])	52 [31.5–75]	68.5 [50–105]	54 [32–103]	70 [47–120]	53 [32–91]	70 [47–111]
Maximum CoP speed, mm/s (median [IQR])	47.5 [41.5–54.5]	65 [58–79]	52.5 [45–73]	64 [55–84]	49 [44–69]	64 [57–81]

**Table 3:** Effects of within-subjects factor (vision: eyes open/eyes closed) and between-subjects factors (physical activity level: low/moderate physical activity; gender: male/female) and the interactions on stabilometric data.

Parameter	Physical Activity	Vision	Gender	Vision × Physical Activity	Gender × Physical Activity
CoP_X_	F = 4.005 *p* = 0.04 η^2^ = 0.05	F = 2.67 *p* = 0.1 η^2^ = 0.03	F = 1.03 *p* = 0.31 η^2^ = 0.01	F = 2.24 *p* = 0.13 η^2^ = 0.02	F = 1.26 *p* = 0.26 η^2^ = 0.01
CoP_Y_	F = 0.31 *p* = 0.57 η^2^ = 0.004	F = 3.44 *p* = 0.06 η^2^ = 0.44	F = 0 *p* = 0.99 η^2^ = 0	F = 0.103 *p* = 0.74 η^2^ = 0.001	F = 2.5 *p* = 0.18 η^2^ = 0.03
CoP displacement	F = 0.13 *p* = 0.71 η^2^ = 0.002	F = 0.13 *p* = 0.71 η^2^ = 0.002	F = 1.95 *p* = 0.16 η^2^ = 0.2	F = 0.17 *p* = 0.67 η^2^ = 0.002	F = 4.06 *p* = 0.04 η^2^ = 0.05
CoP path length	F = 1.404 *p* = 0.24 η^2^ = 0.01	F = 49.3 *p* < 0.001 η^2^ = 0.4	F = 2.55 *p* = 0.11 η^2^ = 0.33	F = 7.9 *p* = 0.006 η^2^ = 0.09	F = 1.02 *p* = 0.31 η^2^ = 0.01
90% confidence ellipse	F = 0.41 *p* = 0.52 η^2^ = 0.006	F = 5.94 *p* = 0.01 η^2^ = 0.07	F = 0.58 *p* = 0.44 η^2^ = 0.008	F = 1.49 *p* = 0.22 η^2^ = 0.02	F = 0.01 *p* = 0.9 η^2^ = 0
Maximum CoP speed	F = 1.03 *p* = 0.31 η^2^ = 0.01	F = 19.78 *p* < 0.001 η^2^ = 0.21	F = 0.74 *p* = 0.39 η^2^ = 0.1	F = 3.14 *p* = 0.08 η^2^ = 0.04	F = 1.64 *p* = 0.2 η^2^ = 0.2
